# Supplementary material for: CHI3L1, NTRK2, 1p/19q and IDH Status Predicts Prognosis in Glioma
Source: Cancers (Basel). 2019 Apr 15;11(4):544. doi: 10.3390/cancers11040544 (PMC6521129; doi:10.3390/cancers11040544)
Supplement: Supplementary file 1 [file cancers-11-00544-s001.pdf]

cancers

MDPI

Supplementary Materials: *CHI3L1*, *NTRK2*, *1p/19q* and *IDH* Status Predicts Prognosis in Glioma

Elise Deluche, Barbara Bessette, Stephanie Durand, François Caire, Valérie Rigau, Sandrine Robert, Alain Chaunavel, Lionel Forestier, François Labrousse, Marie-Odile Jauberteau, Karine Durand and Fabrice Lalloué

Table S1. Clinical and histological characteristics of the French cohort according to molecular group.

Characteristics		Total	Group A	Group B	*p*-Value
N		64	31	33	
Age, years					
	Median	47.5	55.0	40.0	0.0024
	Range	22–81	26–81	22–80	
Sex					
	Female/male	26/38	12/19	14/19	0.7
Surgical approach, *n* (%)					
	Complete resection	29 (45)	11 (35)	18(54)	0.12
	Subtotal resection	35 (55)	20 (65)	15(46)	
Histology, *n* (%)					
	Astrocytoma (II/III/IV)	7/17/21	4/4/17	8/13/4	0.0002
	Oligodendroglioma (II/III)	7/8	2/6	5/2	
	IDH mutant/*IDH* wild-type	36/28	14/17	22/11	0.04
	1p19q codeletion: yes/no	14/50	6/25	8/25	0.6
Localization, *n* (%)					
	Frontal	30 (46)	15 (48)	15 (45)	
	Temporal	19 (30)	7 (23)	12 (37)	0.2
	Parietal	11 (18)	8 (25)	3 (9)	
	Occipital	4 (6)	1 (4)	3 (9)	
Radiotherapy in first line, *n* (%)		44 (69)	25 (80)	19 (63)	0.04
Chemotherapy in first line, *n* (%)		35 (54)	20 (64)	15 (51)	0.1

p-value was calculated for all patients in the histology and localization groups by chi-square test.

Table S2. Clinical and histological characteristics of the TCGA cohort.

Characteristics		Total	Group A	Group B	*p*-Value
Number		671	283	388	
Age, years					
	Median	46.0	58.0	38.0	<0.001
	Range	18–89	21–89	18–87	
Histology					
	Astrocytomas (II/III/IV)	144/186/157	22/90/154	122/96/3	<0.001
	Oligodendrogliomas (II/III)	105/79	7/10	98/69	
IDH mutant/*IDH* wild-type		421/250	53/230	368/20	<0.001
1p19q codeletion: yes/no		181/491	18/265	163/225	<0.001

Table S3. Univariate and Multivariate Cox Proportional-Hazards Models for gliomas in TCGA cohort.

**Characteristics**	**No. of Patients**	**Overall survival**			
		**Univariate**		**Multivariate**	
		**HR (95% CI)**	***p***	**HR (95% CI)**	***p***
**Age at diagnosis**	671	1.067 (1.05–1.07)	<0.0001	1.033 (1.02–1.04)	<0.0001
**Histologic type**					
Astrocytoma	487	Reference	<0.0001	Reference	
Oligodendroglioma	184	0.075 (0.0001- 0.14)		0.775 (0.37–1.59)	0.489
**Grade**					
II	249	Reference		Reference	
III	265	3.185 (2.16–4.69)	<0.0001	1.93 (1.28–2.91)	0.002
IV	157	19.313 (13.01–28.67)	<0.0001	3.65 (2.17–6.14)	<0.0001
**Molecular group**					
Poor prognosis group	235	29.129 (12.80–66.26)	<0.0001	7.55 (2.69–21.20)	0.0001
Intermediate group	355	3.347 (1.455–7.702)	0.004	2.69 (1.07–6.77)	0.035
Good prognosis group	81	Reference		Reference	

Table S4. Custom-made human-specific TaqMan® micro-fluidic cards containing panels of 96 gene expression assays.

Function	Gene Name	Gene Symbol	GenBank No.	Human Assay ID	Function	Gene Name	Gene Symbol	GenBank No.	Human Assay ID
**Receptors**	EGFRa	EGFR	NM_005228	Hs01076078_m1	**Neurotrophin pathways**	NTRK1	NTRK1	NM_001007792.1	Hs01021011_m1
	EGFRb	EGFRb	NM_201282	AI89KGC		NTRK2	NTRK2	NM_001007097.1	Hs00178811_m1
	EGFRc	EGFRc	NM_201283	AIAAZL5		NTRK2	NTRK2 T1 (NTRK2 bis)	NM_001007097.1	Hs01093110_m1
	EGFRD	EGFRD	NM_201284	AIBJXSD		NTRK2	NTRK2 FL (NTRK2 variant)	NM_006180.3	Hs01093096_m1
	EGFRvIII	EGFRvIII	custom-designed	AICSVYL		NTRK3	NTRK3	NM_001007156.2	Hs00176797_m1
	ERB-2	ERRB2	NM_004448	Hs01001580_m1		P75	P75	NM_002507.3	Hs00609977_m1
	ERB-3	ERRB3	NM_001982	Hs00951464_m1		SORTILIN 1	SORT1	NM_001205228.1	Hs00361760_m1
	ERB-3	sERRB3	NM_001005915.1	Hs00176538_m1		NEUROTENSIN	NTS	NM_006183.4	Hs00175048_m1
	ERB-4	ERB4	NM_005235	Hs00955525_m1		NEUROTENSIN RECEPTOR 1	NTSR1	NM_002531.2	Hs00901551_m1
	MET	MET	NM_000245	Hs01565584_m1		NEUROTENSIN RECEPTOR 2	NTSR2	NM_012344.3	Hs00892563_m1
	EGF	EGF	NM_001963	Hs01099999_m1	**Hypoxia**	HIF-a	HIF-a	NM_001243084.1	Hs00153153_m1
**Ligands**	TGF-a	TGF-a	NM_003236	Hs00608187_m1		HIF-b	HIF-b	NM_001197325.1	Hs01121918_m1
	AMPHIREGULIN	AREG	NM_001657	Hs00950669_m1		VEGF-A	VEGF-A	NM_001025366.2	Hs00900055_m1
	EPIREGULIN	EREG	NM_001432	Hs00914313_m1		VEGF-2	KDR	NM_001243733.1	Hs00173634_m1
	BETACELLULIN	BTC	NM_001729	Hs01101204_m1		VEGF-3	FLT4	NM_005429.2	Hs00153458_m1
	HEPARIN-BINDING GROWTH FACTOR	HB-EGF	NM_001945	Hs00181813_m1		VEGF-D	VEGF-D	NM_004469.4	Hs01128657_m1
	NEUREGULIN 1	NRG1-1	NM_001160001.1….	Hs00247620_m1		VEGFR-I	VEGFR-I	NM_001159920.1	Hs01052961_m1
		NRG1-2	NM_001160002.1	Hs01108479_m1		VEGFR-II	VEGFR-II	NM_002253.2	Hs00911700_m1
		NRG1-3	NM_013959.3	Hs01103792_m1		VEGFR-III	VEGFR-III	NM_002020.4	Hs01047677_m1
	NEUREGULIN 2	NRG2	NM_001184935.1	Hs00171706_m1	**Autophagy**	LC3	LC3	NM_032514.3	Hs01076567_g1
	NEUREGULIN 3	NRG3	NM_001165972.1	Hs01377907_m1		BNIP3	BNIP3	NM_004052.2	Hs00969291_m1
	NEUREGULIN 4	NGR4	NM_138573.3	Hs00945535_m1		BECLIN	BECLIN	NM_003766.3	Hs00186838_m1
	TOMOREGULIN	TMEFF- 1	NM_003692.4	Hs00902905_m1		PINK1	PINK1	NM_032409.2	Hs00260868_m1
	TOMOREGULIN (ERB4)	TMEFF- 2	NM_016192.2	Hs01086906_m1		PARKIN	PARK2	NM_004562.2	Hs01038325_m1
	KRAS	KRAS	NM_033360.2	Hs00364284_g1		NIX	NIX	NM_004331.2	Hs01087963-m1
	NRAS	NRAS	NM_002524.4	Hs00180035_m1		ATG5	ATG5	NM_004849.2	Hs00169468_m1
**Intracellular signaling**	BRAF	BRAF	NM_004333.4	Hs00269944_m1		P62	P62	NM_001142298.1	Hs01061917_g1
	PIK3CA	PIK3CA	NM_006218.2	Hs00907957_m1	**Glycosylation**	CHI3L1	CHI3L1	NM_001276.2	Hs00609691_m1

	AKT1	AKT1	NM_004562.2	Hs00178289_m1
	AKT2	AKT2	NM_013987.2	Hs01086102_m1
	AKT3	AKT3	NM_013988.2	Hs00987350_m1
	CDC42	CDC42	NM_001791	Hs00918044_g1
	RHOA	RHOA	NM_001664	Hs00357608_m1
	RAC1	RAC-1	NM_018890	Hs01588892_g1
	INTERNEXIN-A	INA	NM_032727.3	Hs00190771
	IDH1	IDH1	NM_005896.2	Hs01855675_s1
	IDH2	IDH2	NM_002168.2	Hs00158033_m1
**Glioma markers**	GFAP	GFAP	NM_001131019.2	Hs00909233_m1
	NESTINE	NESTINE	NM_006617.1	Hs04187831_g1
	OLIG2	OLIG2	NM_005806.3	Hs00300164_s1
	NEUROFILAMENT	NEFL	NM_006158	Hs00196245_m1
	SYNAPTOPHYSIN	SYP	NM_003179.2	Hs00300531_m1
	CD34	CD34	NM_001025109.1<br>NM_001773.2	Hs00990732_m1
	P53	P53	NM_000546.5	Hs01034249_m1
	NOTCH2	NOTCH2	NM_001200001.1<br>NM_024408.3	Hs01050702_m1
	BDNF	BDNF	NM_001143809.1	Hs03805848_m1
	NERVE GROWTH FACTOR	NGF	NM_002506.2	Hs01113193_m1
	NT3	NT3	NM_001102654.1	Hs00267375_s1
	NTF4	NT4	NM_006179.4	Hs01921834_s1
	KLRC3	KLRC3	NM_002261.2	Hs00749702_s1
	ST3GAL5	ST3GAL5	NM_001042437.1	Hs00187405_m1
	ATHL1	ATHL1	NM_025092.4	Hs00228253_m1
	ST8SIA1	ST8SIA1	NM_003034.3	Hs00268157_m1
	PRUNE2/KIAA0367	PRUNE2/KIAA0367	NM_015225.2	Hs00322421_m1
	GLT25D2	GLT25D2	NM_015101.2	Hs00362851_m1
	GAA	GAA	NM_000152.3	Hs00164635_m1
**Stem cell markers**	CD133	CD133	NM_001145847.1	Hs01009250_m1
	38261	38261	NM_001173531.1	Hs04260367_gH
	SOX-2	SOX-2	NM_003106.3	Hs01053049_s1
	CXR4	CXCR4	NM_001008540.1	Hs00607978_s1
	SSEA1	SSEA1	NM_002033.3	Hs01106466_s1
	NANOG	NANOG	NM_024865.2	Hs04260366_g1
**Housekeeping genes**	HPRT	HPRT	NM_000194.2	Hs02800695_m1
	18S	18S	NR_003286.2	Hs03928985_g1
	BETA 2-MICROGLOBULIN	B2M	NM_004048.2	Hs00984230_m1
	GAPDH	GAPDH	NM_001256799.1	Hs02758991_g1

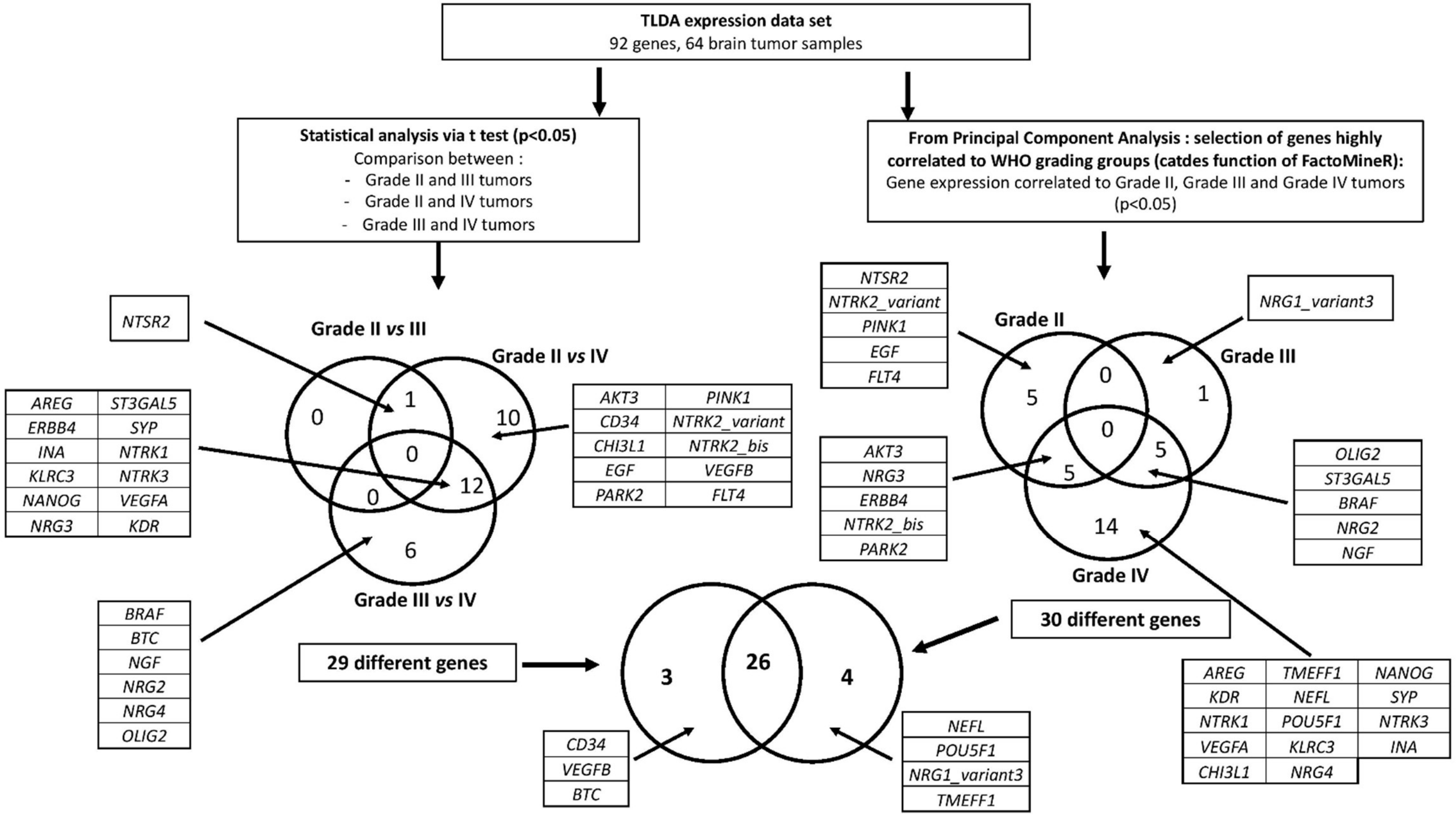

Figure 1. Venn diagram of gene selection in the studied cohort. Intersections between sets indicate the numbers of shared genes, whereas numbers of specific genes are shown in set-specific areas. A total of 92 genes were analyzed from 64 tumor samples, and 26 genes were selected from the cross-section of two statistical methods (*t*-test and principal component analysis).

© 2019 by the authors. Licensee MDPI, Basel, Switzerland. This article is an open access article distributed under the terms and conditions of the Creative Commons Attribution (CC BY) license (http://creativecommons.org/licenses/by/4.0/).
